# On the Use of Large Interactive Displays to Support Collaborative Engagement and Visual Exploratory Tasks

**DOI:** 10.3390/s21248403

**Published:** 2021-12-16

**Authors:** Lei Chen, Hai-Ning Liang, Jialin Wang, Yuanying Qu, Yong Yue

**Affiliations:** Department of Computing, Xi’an Jiaotong-Liverpool University, Suzhou 215123, China; Lei.Chen02@student.xjtlu.edu.cn (L.C.); Jialin.Wang16@student.xjtlu.edu.cn (J.W.); Yuanying.Qu20@student.xjtlu.edu.cn (Y.Q.); yong.yue@xjtlu.edu.cn (Y.Y.)

**Keywords:** interactive displays, user engagement, collaborative learning, large displays, virtual workspaces

## Abstract

Large interactive displays can provide suitable workspaces for learners to conduct collaborative learning tasks with visual information in co-located settings. In this research, we explored the use of these displays to support collaborative engagement and exploratory tasks with visual representations. Our investigation looked at the effect of four factors (number of virtual workspaces within the display, number of displays, position arrangement of the collaborators, and collaborative modes of interaction) on learners’ knowledge acquisition, engagement level, and task performance. To this end, a user study was conducted with 72 participants divided into 6 groups using an interactive tool developed to support the collaborative exploration of 3D visual structures. The results of this study showed that learners with one shared workspace and one single display can achieve better user performance and engagement levels. In addition, the back-to-back position with learners sharing their view and control of the workspaces was the most favorable. It also led to improved learning outcomes and engagement levels during the collaboration process.

## 1. Introduction

Learning in a group allows learners from diverse backgrounds to work together to solve a problem, complete a task, or design an artifact [1,2,3]. Research has shown the benefits of collaborative learning on improving learning outcomes and experiences [4,5,6,7]. Interactive technology, like large displays, can be used to enhance further learning in groups. A large display provides an excellent platform to support group interactions, especially in co-located settings. As they are becoming increasingly pervasive in learning environments, it is important better understand their role in supporting group learning, especially with visual analytical tasks of abstract concepts.

Interactive large displays, such as tabletops [8] and vertical wall screens [9], have been explored in various collaborative settings. These displays allow co-located collaborators to work at different paces and to utilize different exploratory strategies as individuals in their private virtual spaces and as groups in shared spaces. In other words, they can afford different kinds of interactive modes and collaborative couplings when collaborators are co-located physically, more so than desktop computers and mobile devices. Several factors, such as the number of workspaces and displays, shared or non-shared views and control of the content, and position arrangement of the collaborators, can determine how the collaborative process takes place [10]. This, in turn could enhance or impede task performance, knowledge acquisition, and users’ experience with these displays.

Recent research has explored the effect of these factors to help frame the design and use of these displays for co-located collaborative activities [11,12,13]. However, most of this research seems to be focused on sharing one such display for group activities [14,15,16], with limited exploration of multiple displays. Further, it appears the emphasis of this research has been on collaborative drawing or document annotation applications [17], while research focusing on visual analytical tasks with abstract concepts is limited. Having a deeper understanding will allow for the design and deployment of co-located displays to cater to the learning of a broader range of subjects, to support effective modes of teaching/learning that are both cost and space-efficient.

**Figure 1:**
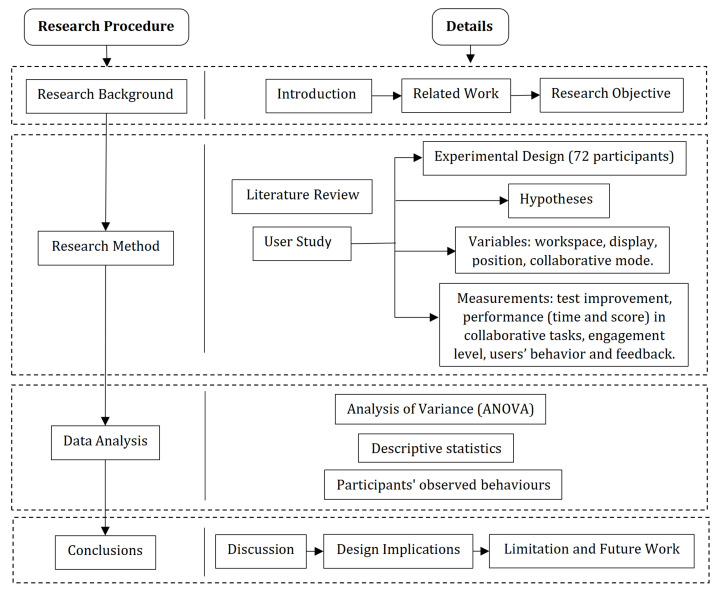
An overview with the main components of this research.

This research attempts to fill this gap and investigates the effect of the number of virtual workspaces, the number of physical displays, the position arrangements of learners, and collaborative modes of interaction on collaborators’ task performance, knowledge acquisition, and engagement level. To do this, we have developed a tool that can support group exploratory tasks with 3D geometrical shapes and run an experiment with four variables: number of workspaces (1 shared or 2 separate), number of displays (1 single or 2 multiple), position of users (Side-by-Side (S-S), Face-to-Face (F-F), Corner-to-Corner (C-C) and Back-to-Back (B-B)), and collaborative modes (shared vs. non-shared view and control of content). Figure 1 shows an overview of this research.

In short, this paper presents three main contributions: (1) An application for large interactive displays that allows users to conduct visual exploratory tasks of the structural properties and transformational processes of 3D geometrical shapes; (2) A user study with the tool to explore the effect of the four variables mentioned earlier on task performance, knowledge acquisition, engagement levels, and collaboration behaviors of users in co-located settings; and, (3) Some implications of our findings for using collaborative visual learning tools and applications in large displays to support positive collaboration in co-located settings.

The remainder of the paper is structured as follows. The following section presents the literature review relevant to this research. The section after describes the collaborative visual tool, including the mathematics background and its visual-interactive features. Then, details about the user study and data analysis are provided in the following section. The session after presents the results and a discussion (see also Figure 1 for an overview of the research).

## 2. Related Work

### 2.1. Collaborative Learning with Large Displays

In collaborative learning, individuals can share information and ideas they explore and discover with others in the group. Computer-supported collaborative learning (CSCL) extends traditional cooperative learning through a technology-enhanced collaborative learning environment, platform, or medium [18,19,20]. Research in CSCL include various types of technologies such as mobile tablets [21,22], large tabletop or wall displays [23], and more recently virtual reality (VR) and augmented reality (AR) [24,25].

Large interactive displays have been shown to improve performance and user satisfaction for tasks such as model design, analysis, and exploration of visual data [26,27,28]. Researchers have developed techniques and interfaces to assist collaborative activities in tabletop environments for group learning [29]. For example, one study with three different conditions (a digital tabletop, personal tablets, and both tabletop and personal tablets) found that the presence of a digital tabletop display improved sensemaking and supported group activities in the shared workspace [30]. Bause et al. noted that a multitouch table with interactive support functions could enhance collaboration and overcome biases from prior preferences [31]. In another study that focused on a face-to-face CSCL environment, Tissenbaum et al. presented the Divergent Collaborative Learning Mechanisms framework. They applied it to support learners shifting between solo and shared work on collaborative activities with tabletops [23].

In the context of collaborative learning with large displays, existing research has explored the design of interface and software supporting learners outcomes [32,33,34] and the arrangement of learning content [35]. Understanding how various system features impact teamwork can better lead to a general improvement of users learning outcomes. Similarly, identifying display factors that can be controlled would allow comparisons across collaborative systems and help elucidate their influence on collaborative processes. However, there is still a gap in the literature exploring how multiple displays and their embedded virtual workspaces, whether assigned to each individual or the whole group, impact the collaborative process and collaborators’ behavior.

### 2.2. Workspace/Display Arrangement and Collaborative Coupling

Technological advances have made possible the provision of multiple large interactive displays to support group activities, including those in educational settings. Some researchers have noted that display arrangements can influence collaborative coupling, which could influence users’ performance and behaviors [36,37,38]. Other researchers have suggested that the number of displays or workspaces can affect users’ position arrangement and collaboration [11,39,40].

Large displays offer the flexibility of supporting multiple workspaces and for collaborators to experience different positions. Previous research suggested that users with a shared workspace can obtain a better shared understanding and more involvement with collaborative tasks than with multiple displays [11,41]. On the other hand, having private spaces, the extreme case being one user assigned to one large display, provides users with more personal space for exploration and hypothesis testing. A study by Inkpen and colleagues [42] showed that group users generally sat closer together with more on-task communication when sharing one display. They also documented users preference for personal workspaces to carry out initial explorations. Another study [43] showed a significant impact of users’ proximity to each other on co-located collaboration in which users shared and interacted with one workspace within a large display. In short, whether providing single or multiple displays and workspaces can affect users’ performance and collaborative behavior. An improved understanding of their effect is helpful for designers and educators to leverage this technology.

Collaborative coupling refers to the position arrangementn of collaborators and the way they interact and communicate with each other [9,16,44]. Liu et al. [45] explored five coupling styles around these displays. Their results showed that providing a shared interaction technique can facilitate collaboration by supporting collaborators to work more tightly even when not in close proximity. Different positions can lead to different user experiences when group work is required [42,46]. For example, learners placed in a face-to-face position can easily view their partners’ face and have eye contact for better communication [46], but they may not have the same perspective of the information unless the two displays are synchronized. Side-by-side positions provide learners with the same perspective but can attract territoriality and privacy issues. One study suggested that users in a side-by-side arrangement found it more effective and enjoyable with a shared perspective. However, users typically had less room and a more obstructed view in the side-by-side configuration [42]. Side-by-side, face-to-face, and right-angle arrangements can be found in the literature, but back-to-back positioning seems underexplored. Back-to-back offers the highest level of privacy. With large displays often considered public platforms, the back-to-back position could mitigate this feeling and lead users to a more focused and efficient exploration. In this research, we plan to assess whether this is the case and explore the relative effect of these position arrangements, including back-to-back, for large displays.

### 2.3. Shared vs. Non-Shared View and Control

Shared virtual workspaces [30,47] allow users to interact with visualized data concurrently. Greenberg [48] has surveyed and discussed systems aimed at providing shared views among distributed worksites. At the time, it represented a paradigm shift in user interface design and emphasized that sharing views and interactions within a single-user application could significantly augment people’s ability to work together. As Isenberg et al. [49] highlighted, co-located synchronous collaboration comes with inherent interaction challenges that arise when multiple people can interact together with the same content synchronously. These interaction conflicts bring to the foreground social norms and conventions that can be difficult to overcome and, when it comes to collaborative learning, it can have unwanted effects. Sharing the same view during collaborative exploration is one possible solution to these issues because, when using multiple displays, a group of learners can have a shared view, as prior research on large tabletops [50] and cross-device displays [51] have shown positive results. This is particularly the case for complex visual problems, as it helps improve awareness of the state of the tasks and can facilitate group communication [52].

In addition to view-sharing, how interaction with content takes place can often affect users behaviors and their predisposition to be involved in the exploratory process. Larsen-Ledet et al. [53] suggested that territoriality issues related to users workspaces could serve as a framework to guide the development of social structures and maintain them during cooperative activities. Some researchers have explored the collaborators’ behaviors with one shared workspace on a tabletop [54] and cross-device workspaces [55]. In addition, others [56,57] have looked at the exploration with two devices that allow users to interact within the same sharing workspace. They point to the importance of examining how interaction should be allowed to take place. In the case of group learning, shared control can be useful to make the process efficient if there is a learner who can drive the process.

Shared control and views for multiple co-located large displays seems underexplored. There is more to be understood about how learners behave in such settings, whether sharing of view and control is beneficial or preferred by these learners, and how their learning and knowledge acquisition can be affected.

## 3. Solid Visualization Tool

**Figure 2:**
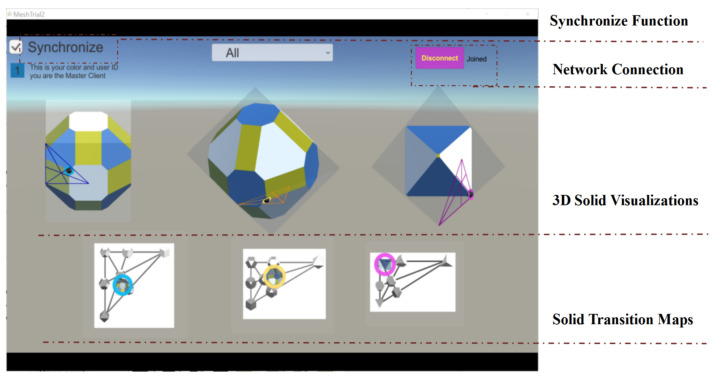
A screenshot of the interface of the 3D solids visualization tool used in this research.

To do our investigation, we have developed a visualization tool to facilitate the collaborative process of exploring, analyzing, and learning 3D geometric shapes (see Figure 2). Peer collaboration is useful for challenges that require spatial reasoning [58]. The visualization tool allows learners to visualize and manipulate abstract geometric structures and their spatial relationships, which is useful to help them with their mathematical reasoning and learning [59,60,61,62]. Their relationships are multilayered, not only dealing with planes of symmetry but also on how the shapes can be derived from each other by truncating/augmenting their vertices and edges.

### 3.1. Visualization Tool Overview

The tool has been developed in Unity3D [63]. As a visualization tool, users can explore and analyze using the tool’s visual interactive functions to interpret the results and draw their own conclusions [64]. No direct or explicit instructions about how to interact with the tool are provided. As shown in Figure 2, the interface of the tool is implicitly divided into four sections: (1) 3D solid visualizations: There are three base solids, cube, tetrahedron, and octahedron (for further details in Section 3.2); (2) Solid transition maps (STM): They can support navigation (movement) within each map and across maps. Within each map, thumbnails of regular solids are connected by lines to indicate the transitional processes of how they can be derived from each other (see Section 3.2); (3) Synchronize function: The transformation of the three solids can be synchronously-linked, and as such changes in one solid are reflected in the others (more details in Section 3.3); and (4) Network connection function: to allow users to switch between Shared and Non-Shared modes by clicking the “Connect/Disconnect” button (see details in Section 3.4).

### 3.2. Interactive Morphing of the Solids

The 3D solid visualizations placed in the middle of the interface present structural information of the solids. Users can learn about the individual shapes and how they can be morphed into other shapes in an interactive way. Each solid is rendered with three different colors indicating the process of truncating their vertices and/or edges (blue is used to show truncated vertices, yellow truncated edges, and white the original faces). The tool provides users with two methods to morph the solids dynamically: direct and indirect manipulations.

**Figure 3:**
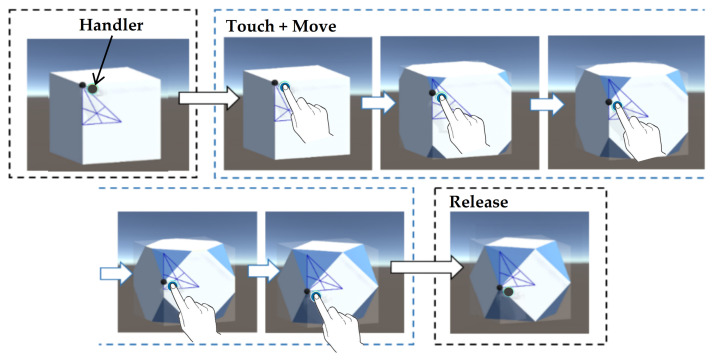
Screenshots of interactive morphing of the shapes: A user uses the handler on the Cube and moves it around until reaching the desired object of exploration. Releasing the handle signals the end of the process.

**Figure 4:**
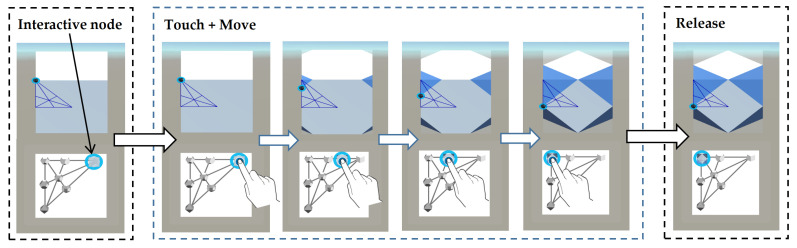
Screenshots of morphing the shapes: A user touches the interactive node in the map and moves it around until reaching the desired object of exploration, which ends when the user releases the node.

**Figure 5:**
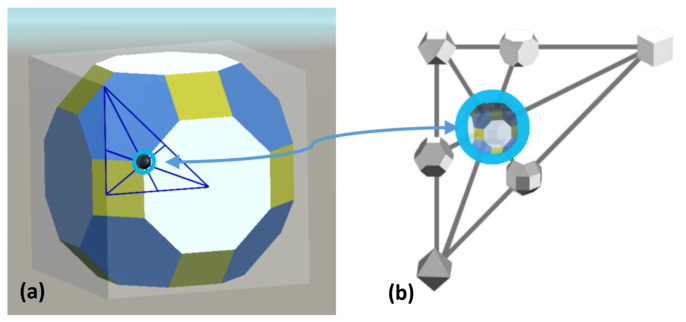
(**a**) Rhombi-truncated cuboctahedron obtained by truncating all the vertices and edges of a cube; (**b**) Solid transition cube-map indicating the current state of the transformed solid.

(1) Direct manipulation. Users can directly control the the handler (the black dot) on the solids and move it around to reach the desired object (see Figure 3). Direct manipulation gives a more direct engagement with an visual object [60,65,66]. The blue colored sides show the vertices that have been cut off (or truncated). On a corner of each base solid, there is a modified solid transition map that shows the current stage of transformation of this solid. The truncating/augmenting operations of users on the solids are constrained in the area of the map. This means that users can control the black dot handler on the solids and move it around on the map on the corner of each solid.

(2) Indirect manipulation. Users can dynamically and continuously morph or transform a 3D solid via the Solid Transition Maps (STM) displayed below each base solid (see Figure 4). Each map has an interactive selected node. Users can interact with this selected node and move it anywhere on the map. There is an implicit link between each base solid and its corresponding map located below it. Figure 5 shows an example of this relationship: the cube with the map attached to the top-right corner (left) and its transition map (right). Each node on the map (Figure 5) represents a possible transformation of the Cube. The interactive node is in the highlighted circle on the map. All changes are reflected on the solid. This interaction is referred to as “indirect manipulation”, where users communicate with an object via a second object [67,68].

### 3.3. Dynamic-Linking of Multiple Visualizations

A twin solid is a shape that has the same structural properties as the current selected solid, but it is different in the way it was obtained [59]. Such property represents more abstract complex connections between multiple STMs. Identical shapes can be obtained from different base solids but through different transformation processes. In our tool, for any selected solid, corresponding twin solids are also shown and highlighted on the other two maps.

**Figure 6:**
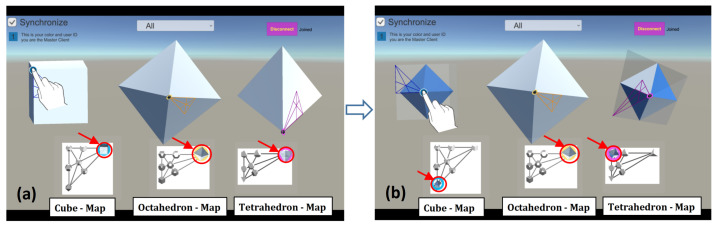
Screenshots of synchronization function: (**a**) One user manipulates the cube to get an octahedron shape; and (**b**) when it reaches the desired object, the same resulting object is displayed in the other solids (i.e., the octahedron) as the visual elements are dynamically linked (in our case implemented as synchronization).

As shown in Figure 6, when users manipulate one of the three solids to obtain the octahedron shape, the other two solids are synchronized to show the same resulting shape (the transformed ctahedron). For example, when one user manipulates the cube to derive the Octahedron (see the Cube to the Octahedron in Figure 6), the effects are shown on the other two solids, because they are are synchronized. The end result is the octahedron, which is shown in all three base solids. Dynamically-linking implemented in our tool can facilitate global knowledge acquisition across multiple maps—-that is, the solids and the maps become dynamically linked to help visualize the existence of the same shapes (i.e., twin solids). Users can switch off the synchronization (using the “Synchronize” toggle located on the top-left corner of the tool) to turn off the dynamic linking of visualizations.

### 3.4. Shared and Non-Shared Control and View across Multiple Displays

As mentioned before, the application has two collaborative modes, Shared and Non-Shared control and view.

**Figure 7:**
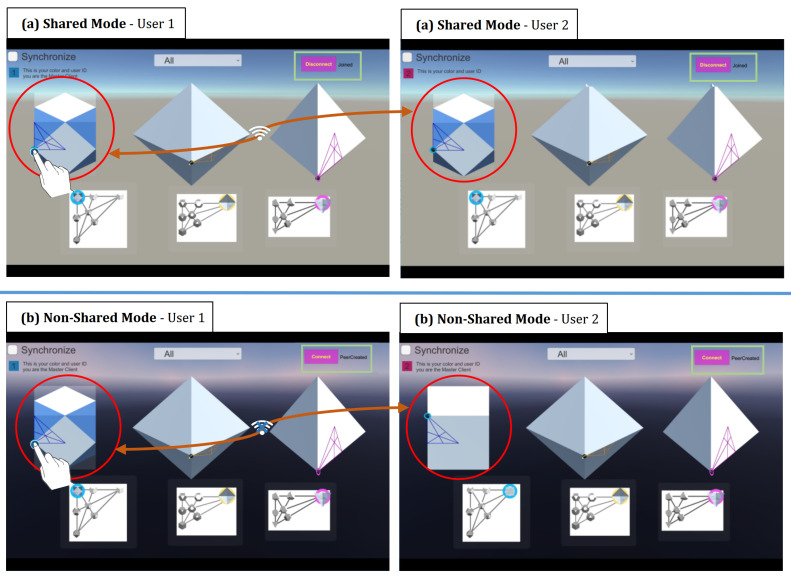
Two different modes of interaction: (**a**) Interaction in the Shared mode (changes in one display are also shown in the other); (**b**) Interaction in the Non-Shared mode (the two displays are disjoint, so changes in one display are not reflected in the other).

(1) In the Shared mode, it allows two (or more) users to interact simultaneously with shared views and to control the visual objects via displays that are connected with each other. As such, changes and interactions carried in one display are reflected immediately in the other display. For example, as can be seen in Figure 7 (top row), the rotation and morphing of the solids in User 1’s display are reflected in User 2’s display. In this way, a user can always see what the partner is exploring by looking at their own display (but they can do so if they still want to). To avoid confusion, each visual element is designed so that only one user can manipulate it at a time. That is, a solid currently being controlled by one user is no longer interactable for the other user. For example, when User 1 interacts with the cube, the cube on User B’s display cannot be manipulated until User 1 releases control over it.

(2) In the Non-Shared mode, the two displays or applications are not linked. As such, changes in one display (like their rotation or transformation) are not reflected on the corresponding solids in the other user’s application (see Figure 7, bottom row). In other words, a user cannot know what the partner is doing unless the user turns his/her head physically and looks at the partner’s application.

## 4. User Study

### 4.1. Experimental Design

**Figure 8:**
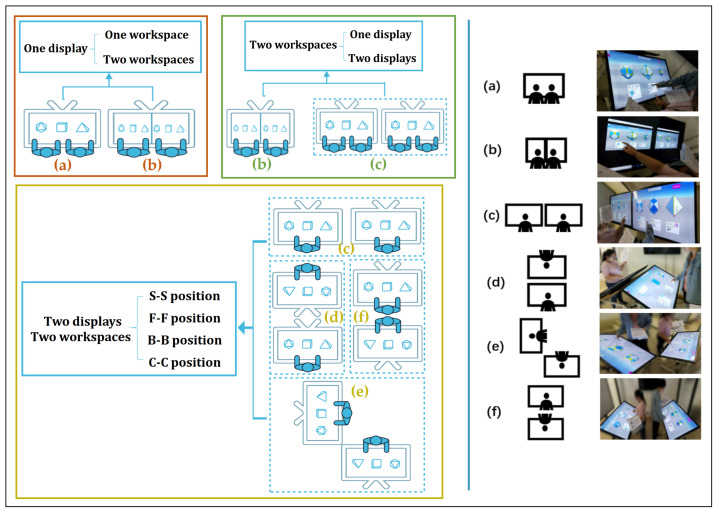
The three kinds of comparisons based on the number of workspaces and displays, and position arrangements: (**a**) One workspace and (**b**) Two workspaces in one display (see Red box); (**b**) One display and (**c**) Two displays (see Green box); and (**c**) Side-by-Side (S-S), (**d**) Face-to-Face (F-F), (**e**) Corner-to-Corner (C-C) and (**f**) Back to Back (B-B) (see Yellow box).

Prior research has shown that large interactive displays can support group activities [26,27]. Some researchers have noted that display arrangements can influence collaborative coupling, which in turn can also affect users’ performance and behaviors [39]. Other researchers have suggested that the number of displays or workspaces can result in different position arrangements and collaboration experiences of users [11]. Therefore, to explore knowledge acquisition, task performance and engagement level of learners using large displays, we explored four factors, the number of workspaces, the number of displays, user position arrangements, and collaborative modes. Based on the first three factors (workspace, display and position arrangement), we have three kinds of comparisons:

(1) Workspace: comparing one and two workspaces. We compared users having one shared workspace (see Figure 8a) and users having separate workspaces, one for each user (see Figure 8b) within one display (see the Red box in Figure 8).

(2) Display: comparing one and two displays. When each of users has a separate workspace, this could be divided into two conditions: (1) two users share one single display (see Figure 8b), and each user has a single display (see Figure 8c), that is, each user would have a separate workspace in his/her own display (see the Green box in Figure 8).

(3) Position arrangement: comparing Side-by-Side (S-S), Face-to-Face (F-F), Corner-to-Corner (C-C), and Back-to-Back (B-B) positions. With multiple displays, each user in a pair would have a single private display and workspace. Therefore, for two displays, we explored how paired users placed at various positions affect their task performance and experience. We considered four positions, S-S, F-F, C-C, and B-B as shown in Figure 8c–f (see the Yellow box in Figure 8).

Besides, as we mentioned earlier, our tool could support shared and non-shared interaction modes. During collaboration, each pair is allowed to switch between these two modes freely. We recorded the time that users in different conditions spent on each mode and their behaviors. According to the time spent, we could categorize their interaction into four types: Shared mode at all times (S), Non-shared mode at all times (NS), more Shared time when switching between modes (SNS), and more Non-shared time when switching between modes (NSS). This classification would allow us to find insights on whether the display factors would affect the choice on the two collaborative modes.

### 4.2. Hypotheses

Based on the experimental design, we aimed to test the following four hypotheses in this experiment:

**Hypothesis** **1** **(H1).** *Sharing one workspace would lead to higher user performance and experience than using two separate workspaces. Users with their individual workspace may engage more in their own thinking process. In a shared workspace, users would be encouraged to engage with each other and pay more attention to collaborating with their partners. Therefore, we expected that users would perform better with one shared workspace.*

**Hypothesis** **2** **(H2).** *Sharing one display would lead to higher user performance and experience than using multiple displays. For paired users, sharing one display would give them a higher collaboration experience. In this condition, users may feel more engaged with each other when completing the tasks. Therefore, we anticipated that paired users would perform better with one shared display than multiple displays.*

**Hypothesis** **3** **(H3).** *Side-by-Side position would be most suitable for improving task performance and engagement with each other. Users could communicate more efficiently and share their ideas with lower effort (or movement) because of their close proximity to each other, which would enhance task performance. Earlier research has shown that users placed side-by-side achieved the best task performance when collaborating with mobile devices [22]. Therefore, we expected that the same result could apply to large displays.*

**Hypothesis** **4** **(H4).** *Users would spend more time on the Shared mode than the non-shared mode. Especially for the B-B position, users would be more dependent on the tool’s shared view and control. Prior research emphasized the importance of collaboration in supporting learning. We expected that with large displays, users would be more engaged in the shared mode. It could facilitate sharing ideas while solving problems.*

### 4.3. Participants

Seventy-two students (38 females and 34 males) aged between 18–27 (M = 20.37, SD = 2.64) were recruited to participate voluntarily in this study. We advertised our study through email listservs and via internal online forums and social media platforms at a local university to ensure that participants had diverse educational backgrounds. None of them had used the tool before the experiment. Among the 36 pairs, only three pairs did not know each other before participating in the experiment. All participants had normal or corrected-to-normal vision and had no issues distinguishing the colors we used in the application. We collected their demographic data and spatial reasoning skills and collaboration preferences via a pre-experiment questionnaire. The data showed that 60% of the participants believed they had good spatial reasoning skills, and 84.29% thought of themselves as good at cooperating with others. They reported an average of 3.29 and 2.47 respectively for spatial reasoning skills and collaboration ability (with 1 = very bad, 2 = bad, 3 = neutral, 4 = good, and 5 = very good).

We followed a between-subjects design to avoid carry-over learning effects, where one participant could only experience one condition. We also pre-screened the participants to ensure they had not interacted with the tool before. The participants were paired, and each pair was randomly assigned to a condition to complete the same set of tasks (see Table 1). In the end, there were six groups assigned to each condition.

### 4.4. Apparatus

The experiment took place in a dedicated lab room where each pair could freely communicate (see Figure 8). The 3D solid application was run on a 50-inch TV with 4K resolution (Xiaomi, Beijing, China) and multitouch capabilities, and was connected to a desktop running Windows with an i7 GPU, GeForce GTX 1080 GPU, and 16 GB of memory (NVIDIA, Santa Clara, CA, USA). The TV displays were positioned at a slight inclination (about 30 degrees) to provide a comfortable view and interaction. Touch interaction was the primary way for participants to interact with the tool.

During the experiment, each pair stood next to their assigned display according to their condition. We used a video camera to capture participants’ interactions with the tool and their verbal and non-verbal communications for later analysis. The interactions were also screen-captured for later assessment.

### 4.5. Tasks and Procedure

#### 4.5.1. Pre- and Post-Experiment Test

**Figure 9:**
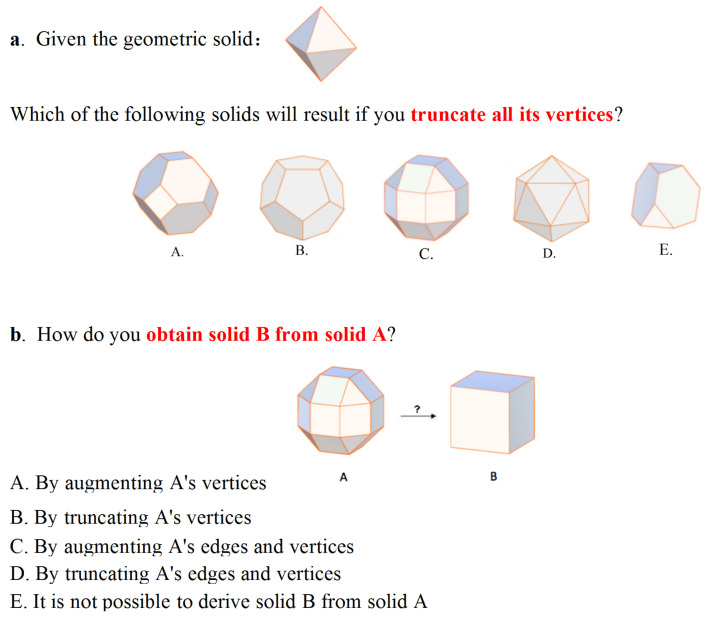
Two sample questions used in the pre- and post-test.

To determine knowledge improvement before and after interacting with the tool, participants were required to complete a test before engaging with the tool and the same test after. The test consisted of 12 questions based on the transformational and structural properties of the solids. Participants were asked to select the correct answer among several possible choices. Figure 9 shows two sample questions. For example, Question b asks participants to determine what interaction operations (truncating or augmenting) are required to obtain Solid B from Solid A. Each question, when answered correctly, was given one point (that is, 12 points in total). Two users were required to complete the test independently without any communicating with each other.

#### 4.5.2. Collaborative Tasks

**Figure 10:**
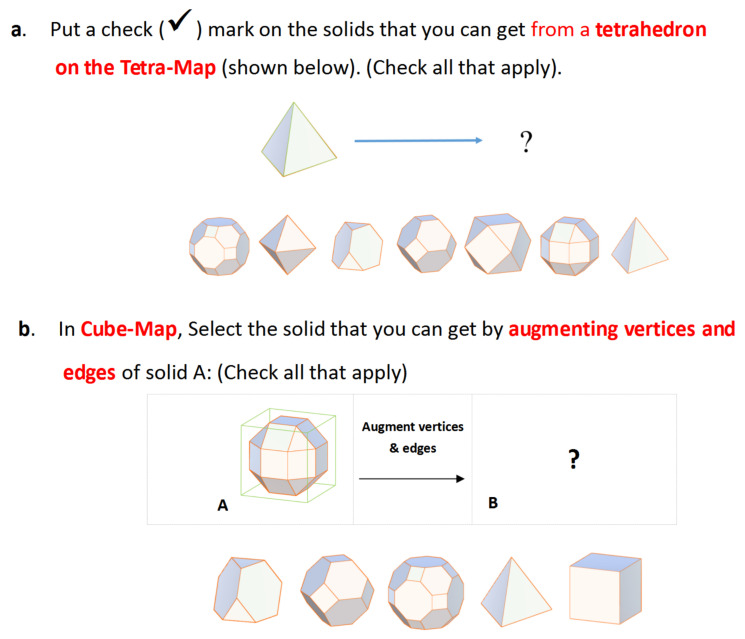
Two sample collaborative tasks used in the study.

During the experiment, participants were given a set of exploratory, question-based tasks to complete with the help of the tool. The tasks were intended to provide participants with predetermined goals to facilitate data gathering. They were encouraged to collaborate and talk with each other during the exploration process. The tasks would require participants to perform the interactive operations with the shapes. Figure 10 shows two example tasks. For example, Question a asks participants to determine which is the resulting shape among the seven choices when the base shape’s vertices and edges are truncated. Participants were asked to choose all transformed shapes in the below options. The details of the tasks were provided on paper to each participant. Each pair completed the tasks together based on the condition to which they were assigned.

#### 4.5.3. Procedure

Each session was divided into seven phases: (P1) Informing participants of the purpose of the study and the ethics regulations governing it, and then completing the consent form plus a short questionnaire to collect anonymized demographic data (∼5 min); (P2) Pre-test (see the details in Section 4.5.1) with 12 knowledge-based questions about the geometric shapes within 10 mins. If users cannot finish all tasks within the time limit, the tasks that they did not complete would be given 0 points; (P3) A training session was provided to participants to help them become familiar with the interface and interactive features (∼5 min). (P4) Performing 12 predefined collaborative tasks (see the details in Section 4.5.2) with the tool. Each pair was asked to give an agreed-upon answer—that is, the pair must agree with the answer to each question by communicating and discussing their results. During collaboration, participants were allowed to freely switch the shared and non-shared modes based on their preference. For shared mode, each solid was designed so that only one user could manipulate it at a time. They needed to negotiate who would do the manipulations (∼25–30 min); (P5) Completing the engagement questionnaire (∼5 min); (P6) Performing post-test which was the same as the pre-test (10 min); and (P7) A brief interview on their experience of the experiment (∼5 min). The whole experiment took about one hour for each pair.

### 4.6. Data Analysis

In this study, we employed both quantitative and qualitative measures. We used quantitative analysis to quantify users’ knowledge acquisition of the domain. Therefore, we measured the test improvement scores, correct scores and completion time of the collaborative tasks. For the pre-test, users answered the questions based on their previous knowledge, if any. Then, we would evaluate users’ completion time in and correct answers to the collaborative tasks. Finally, after interacting with the tool, the post-test scores would measure the knowledge gained after their interactions. Overall, these objective data can help us to measure the learning outcome of users during this study. In addition, data from a post-experiment questionnaire that contained 7-point Likert-scale questions would allow us to quantify participants’ perceived level of engagement and collaboration experience. We also analyzed the videos of participants’ interactions to assess their collaboration behaviors (that is, how pairs in each condition tended to work together and interact with the tool).

One-way ANOVA tests were used to determine any differences in the data using an alpha value of 0.05 to determine the significance level. Bonferroni corrections were used in post-hoc analysis.

We next present the results of quantitative measures first and then combine those with the qualitative measures to cross-validate the data, particularly for user performance and collaboration patterns. M, SD, and SE are used to denote mean, standard deviation, and standard error values for simplicity.

## 5. Results

Given that we have 4 factors (workspace, display, position arrangement, and collaborative mode), the presentation of the results is organized based on 4 categories. Table 2 shows a summary of the main results of the statistical analysis.

### 5.1. Pre- and Post-Experiment Test

Figure 6a shows the distribution of the scores of the pre- and post-experiment tests based on the different conditions. One-way ANOVA yielded no significant differences (F${}_{1,22}$ = 0.298, *p* = 0.590, η${}_{p}$${}^{2}$ = 0.013, observed power = 0.082) on test scores between one shared workspace (M = 0.417, SD = 1.564, SE = 0.432) and two separate workspaces (M = 0.750, SD = 1.422, SE = 0.432). There was no significant difference (F${}_{1,22}$ = 0.015, *p* = 0.903, η${}_{p}$${}^{2}$ = 0.001, observed power = 0.052) between one single display (M = 0.750, SD = 1.422, SE = 0.411) and multiple displays (M = 0.667, SD = 1.875, SE = 0.541). For the position arrangements, although participants in B-B achieved the highest scores (M = 1.167, SD = 2.406, SE = 0.694), and F-F the lowest (M = 0.500, SD = 2.067, SE = 0.597), we did not find any significant difference in scores based on positioning.

### 5.2. Completion Time and Scores on Collaborative Tasks

**Figure 11:**
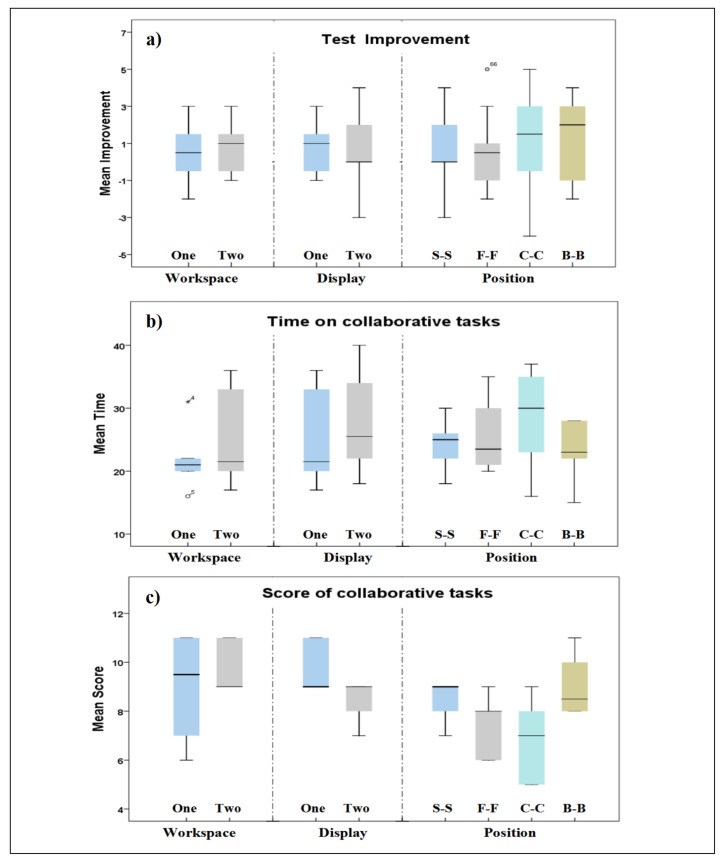
Plots of score improvement between pre- and post-experiment tests (**a**); time spent on collaborative tasks (**b**); score on experiment tasks (**c**); S-S: Side-by-Side, F-F: Face-to-Face, C-C: Corner-to-Corner, B-B: Back-to-Back.

Completion time and scores would allow understanding how efficiently participants collaborated in the different conditions. We recorded the scores and the time for completing the 12 tasks and observed participants’ behaviors. Figure 11b,c show the distribution of time and scores for the experiment tasks.

Workspace. Paired users with one shared workspace spent less time than two separate workspaces (see Table 3). However, they achieved slightly lower scores with one shared workspace than two separate workspaces. There was no significance on time (F${}_{1,10}$ = 0.558, *p* = 0.472, η${}_{p}$${}^{2}$ = 0.060, observed power = 0.112) and on score (F${}_{1,10}$ = 0.488, *p* = 0.501, η${}_{p}$${}^{2}$ = 0.047, observed power = 0.097) between one shared workspace and two separate workspaces.

Display. Users sharing one single display spent less time in completing the collaborative task but still achieved slightly higher mean scores than pairs using multiple displays (see Table 3). A one-way ANOVA test yielded s significant difference on time (F${}_{1,10}$ = 4.623, *p* = 0.047, η${}_{p}$${}^{2}$ = 0.316, observed power = 0.493) but not on scores (F${}_{1,10}$ = 0.341, *p* = 0.572, η${}_{p}$${}^{2}$ = 0.033, observed power = 0.083) between one single display and multiple displays.

Position. Participants placed in B-B achieved the highest average scores and spent less time, followed by S-S and F-F. Those who were in C-C had the lowest performance (see Table 3). A one-way ANOVA test yielded a significant effect of position on time (F${}_{3,20}$ = 3.586, *p* = 0.032, η${}_{p}$${}^{2}$ = 0.350, observed power = 0.703). Further pairwise analysis showed that B-B was more efficient than C-C (*p* = 0.045). No significance was found for the other groups. We did not find any significance on score among the different positions (F${}_{3,20}$ = 0.866, *p* = 0.475, η${}_{p}$${}^{2}$ = 0.115, observed power = 0.204).

### 5.3. Perceived Engagement Levels during Collaboration

The engagement level effectively reveals learners’ persistence, performance, and satisfaction with their learning [69,70]. A 7-point Likert scale user engagement questionnaire was used to measure participants’ engagement levels based on seven categories [71]. The questionnaire for assessing collaboration experience was derived from the work of Isenberg et al. [16] and Jakobsen and Hornbæk [9]. After making some adaptations, our final questionnaire presented 23 questions (see Appendix A).

**Figure 12:**
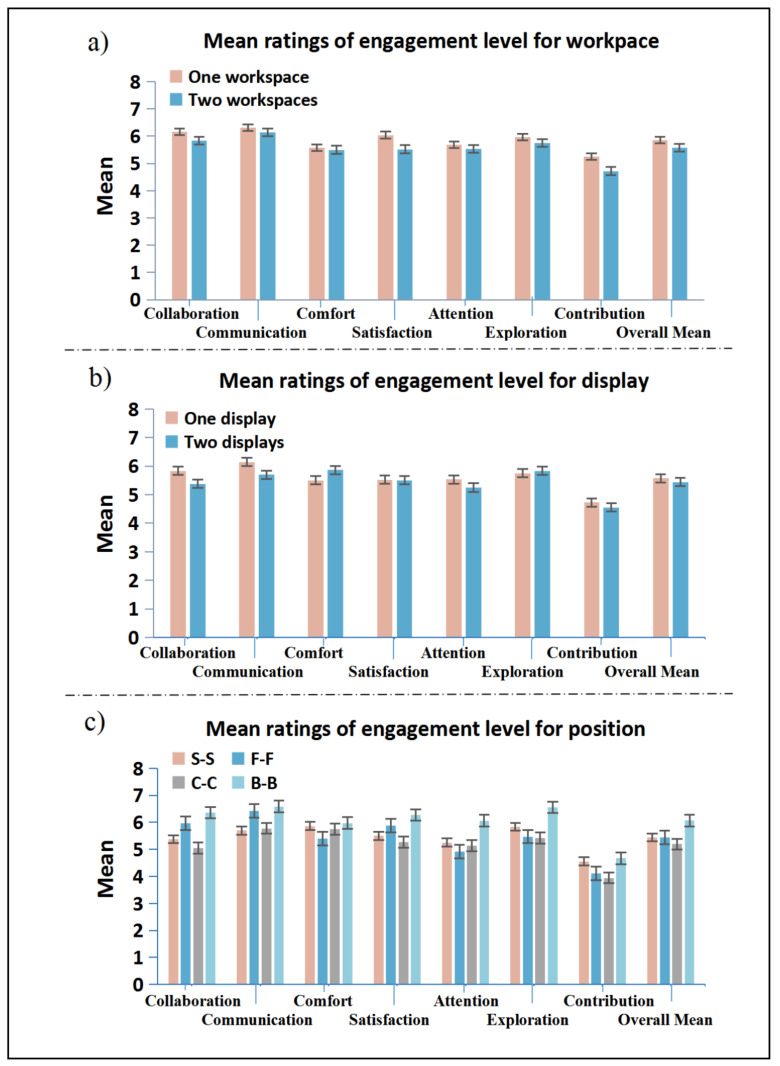
Plots of mean ratings of engagement level for workspace (**a**), display (**b**), position (**c**); C-C: Corner-to-Corner, F-F: Face-to-Face, S-S: Side-by-Side, B-B: Back-to-Back.

Overall engagement ratings. As shown in Figure 12a, users with one shared workspace obtained a slightly higher overall level for engagement (M = 5.859, SD = 0.583, SE = 0.168) than the pairs with separate workspaces (M = 5.570, SD = 0.356, SE = 0.103). From Figure 12b, we can also observe that the one single display pairs (M = 5.570, SD = 0.356, SE = 0.103) provided slightly higher ratings than the pairs with multiple displays (M = 5.439, SD = 0.683, SE = 0.139). Despite these results, a one-way ANOVA yielded no significance of display (F${}_{1,22}$ = 0.216, *p* = 0.646, η${}_{p}$${}^{2}$ = 0.010, observed power = 0.073) and workspace (F${}_{1,22}$ = 2.143, *p* = 0.157, η${}_{p}$${}^{2}$ = 0.089, observed power = 0.288) on overall engagement level.

Based on their position arrangement, we can clearly observe that participants in B-B gave higher scores (M = 6.065, SD = 0.510, SE = 0.147) on overall engagement level than the other three positions. S-S (M = 5.438, SD = 0.916, SE = 0.264) and F-F (M = 5.448, SD = 0.744, SE = 0.215) received similar scores (see Figure 12c). C-C was ranked the lowest (M = 5.192, SD = 0.786, SE = 0.227). A one-way ANOVA yielded a significant difference on overall engagement level for position (F${}_{3,44}$ = 2.935, *p* = 0.044, η${}_{p}$${}^{2}$ = 0.167, observed power = 0.658). Post-hoc pairwise comparisons showed a significant difference on overall engagement between B-B and C-C (*p* = 0.041).

Ratings on the engagement categories. For the engagement subscales, one workspace received higher scores than two workspaces on all subscales (see Figure 12a). Besides, participants with one single display gave higher scores on Collaboration, Communication, Satisfaction, Attention, and Contribution subscales than those with multiple displays. Participants may have a stronger preference for sharing and communicating when working with one shared workspace or one single display. However, we found that participants using multiple displays provided higher scores on Exploration and Comfort. A one-way ANOVA test yielded no significant difference of display on any subscales (all *p* > 0.05). For the Satisfaction subscale, there was a near significance (*p* = 0.066) between one shared workspace (M = 6.042, SD = 0.789, SE = 0.228) and two separate workspaces (M = 5.521, SD = 0.494, SE = 0.143).

In terms of the positions, we can see that B-B obtained the highest ratings on all subscales compared with the other positions (see Figure 12c). We can also see that the range was smaller. A one-way ANOVA test yielded a significant effect of the different positions on Collaboration (*p* = 0.009), Satisfaction (*p* = 0.037), Exploration (*p* = 0.016) and a close to significant effect on Attention (*p* = 0.054). Post-hoc pairwise comparisons revealed that B-B was significantly better than C-C on Collaboration (*p* = 0.012), Satisfaction (*p* = 0.042), Exploration (*p* = 0.025). In addition, B-B also received significantly higher ratings than F-F on Exploration (*p* = 0.038). There was no significance on other subscales for the other conditions (all *p* > 0.05). It seems clear that participants in B-B perceived a higher engagement level than those in the other positions, significantly higher than C-C and F-F.

### 5.4. Participants’ Preference on Shared and Non-Shared View/Interaction

As stated earlier, each pair was allowed to freely switch between shared and non-shared modes of collaboration. Excluding the six pairs who worked in the one shared workspace in the single display condition, we analyzed the preference of the other 30 groups with two modes while completing the collaborative tasks. Based on the results, in general, more participants preferred switching between the two modes (53%) instead of being in S mode (30%) and NS mode (17%). For participants only using one mode during the experiment, S mode was more preferred than NS mode. Further, shared view and control (S mode and SNS mode, 60%) was the more popular choice. In other words, for one single display with two separate workspaces, more users (67%) chose the NS mode, and 33% of them switched between modes and spent more time on NSS mode. Because they shared one display that allowed ease of view of the tool, no one chose the S mode and SNS mode. When using multiple displays with two separate workspaces, participants with different position arrangements had different preferences. In the S-S position, 17% of them kept using NS mode only, and 83% switched back-and-forth based on their preference (33% for SNS mode and 50% for NSS mode). For the other positions, 33% of participants in F-F preferred S mode, and 67% chose the two switching modes (50% for SNS and 17% for NSS). In C-C position, 50% were in the S mode, and 50% preferred the two switching modes (33% for SNS mode). Finally, for B-B position, 67% chose S mode while 33% SNS mode. The details are shown in Table 4.

### 5.5. Subjective Feedback

Position preference. Because we followed a between-subjects design, each participant had to complete the set of tasks at one position only. After the experiment, we also collected their preferences on different positions if they could choose a position. A slightly higher number of participants chose B-B (38.33%), followed by S-S (33.89%), F-F (16.67%), and C-C (11.11%).

Collaboration experience. Responses to the questions about their collaboration experience showed that 69.45% of participants thought that the relationship among users would affect learning performance. 73.60% said they were interested in the collaborative mode of learning with large displays, and 76.39% thought collaborative learning is more helpful in improving learning efficiency than independent learning.

## 6. Discussion

### 6.1. Workspace(s)

We explored whether the number of workspaces could impact users’ performance and collaborative behaviors. The workspace(s) in one shared display could lead to territoriality issues in collaborative settings [53,54,72]. From our results, we found that paired participants with one shared workspace performed better on mean task efficiency than two separate workspaces when completing the collaborative tasks, which partially aligns with **H1**. With one shared workspace, participants seemed to feel that they were closer to each other. They did not have to move much to share their control and ideas, which was helpful for saving time and improving the task efficiency. Although there was no significance of workspace on task scores (test improvement and collaborative task scores), we still found that using separate workspaces for each user may have helped users to obtain a higher task score—this result conflicts with our

textbfH1. This result seems to imply that shared workspace may have a negative effect on participants’ outcomes when working together.

Concerning engagement level, participants rated one shared workspace higher than two separate workspaces on overall engagement level and its subscales, especially on Satisfaction and Contribution. This result supports our **H1**. From this result, we can conclude that a shared workspace may let users be more engaged during collaboration. They would feel more satisfied with the shared workspace when doing collaborative tasks. In addition, participants talked more and shared ideas more frequently in shared workspace settings. One user mentioned that “with a shared workspace, it is very helpful for enhancing collaboration especially for shy users”. Because they were not very active during collaboration, they would engage less in the collaboration process when they had a personal workspace. In a shared workspace, users would be encouraged to talk more with each other. They would have more communication and interaction because of their short distance from each other. This finding is supported by the previous research [11,41], which has suggested that with a shared workspace, users can have a better shared understanding and more involvement in collaborative tasks. We also observed that users shared their ideas with the partner more frequently by showing the operations in the application. They often used ’look’, ’this’ and ’by doing it in this way’, etc. when trying to share their ideas and findings with their partner. This active interaction with each other, in turn, led users to be more engaged.

In addition, one participant mentioned that when using two separate workspaces with a single display, the size of this display seemed somewhat small. The display in our study, even though it was a 50-inch TV, was in his view not big enough to have two workspaces. In this case, as some solids in the application were a little small, it would have been better to have only one single workspace in one display. From this feedback, we should also consider the balance between the size of the display and the workspace, and the quantity of visual elements shown. This is a noteworthy point when designing and using collaborative displays.

Based on our above observations, although there is some degree of support for **H1** from the results, they are not conclusive. Therefore, it is not supported.

### 6.2. Display(s)

This experiment explored whether the number of displays (single and multiple) would affect users task performance and learning efficiency. Based on the results, we found that the number of displays has an influence on the users’ performance in collaborative learning scenarios. Specifically, participants (in pairs in our study) using one display achieved higher scores on their post-test. In addition, one single display led to statistically better performance and less time than multiple displays on the collaborative tasks, which strongly aligns with **H2**. That is, participants spent less time and still achieved higher scores on the tasks they completed together. From this result, we can find that using one single display seems to be helpful for improving task efficiency and performance when collaboration is needed. This result would imply that sharing one large display (50-inch TV in our study) might be better than using multiple displays in collaborative learning settings when doing visual analytical tasks, like those in our experiment. This is in line with previous research [11] which shows that a shared artifact can help foster a better shared understanding than multiple displays. In terms of participants’ behaviors, we observed that they generally stood closer together and exhibited more communication and interaction with each other when they shared one display. Furthermore, when they shared one display, it was more convenient to share the ideas and operations with their partner although they still used separate workspaces, which enhanced the collaboration process and decreased time. Overall, sharing one display when collaborating required less time and could improve task performance with higher learning outcomes. Research reported in [73] also stated that the around-the-table setup was beneficial for collaboration, which also supports our findings.

In terms of engagement level, participants with one single display achieved higher scores on the overall engagement level. These participants felt more engaged when using one display during their collaboration. With one single display, they were stood closer to each other, which also facilitated communicating and sharing their ideas. For individual subscales, we found that users with one display gave higher scores on Collaboration, Communication, Satisfaction, Attention, and Contribution than participants using multiple displays. These results align with our **H2**, and also were supported by the findings from [74], which suggest that sharing a single display can increase attention and involvement in collaborative tasks. The results in [42] also showed that a group of users generally sat closer together with more communication about their tasks when sharing one display. Some participants mentioned that when they had a personal display, the frequency of communication could be reduced, even if the distance between them was short. However, we found that multiple displays received higher scores on Exploration and Comfort subscales. This result suggests that a personal space could lead to more private exploration, something that was not conducive when the display was shared. When given a personal display, users might have more time to explore on their own when completing the tasks. Besides, participants having their own single display would feel more comfortable and in control. In one study, the authors stated that monitoring was easier when sharing a display, which would facilitate the coordination of action in a shared task [75]. However, we observed that users with individual displays tended to use less body language and communicate less. Some users with separate displays said they felt that they had personal space and thereby felt safer.

According to the above discussion, **H2** seems to be supported by the results and therefore can be accepted.

### 6.3. Position Arrangement(s)

Based on the results, participants placed in B-B positions achieved higher improvements on test scores. It is also worth mentioning that participants in B-B completed the tasks collaboratively using the shortest time but still achieved higher scores compared to the other positions, especially than those in C-C. This suggests that B-B positions could lead to better learning outcomes for collaborative tasks than C-C ones. Therefore, B-B seems to be the most suitable in this kind of learning scenario, which contradicts our **H3**. We thought that S-S would be better than the other single positions. However, based on our results in this study, we found that B-B was the best one. This result also conflicts with the findings in some prior works (e.g., [3,22]). Their results showed that S-S was the best position for supporting better task performance. It is interesting to see that B-B led to the best performance (i.e., the highest average score and lowest performance time), then followed by S-S, F-F and C-C. On the other hand, we observed the reversed order (i.e., C-C, F-F, S-S, and B-B) in time spent on the tasks by participants. These two observations could help researchers and instructors to consider which arrangement(s) should be used based on their priorities.

In addition, we found that B-B also received the highest scores on engagement level compared to the other positions, both overall level and subscales. According to the results, participants perceived to be more engaged while in B-B than in C-C. This finding does not align with **H3** and as such it cannot be accepted based on our results. Furthermore, B-B was rated significantly higher on Collaboration, Satisfaction, and Exploration. As such, it could be inferred that participants in B-B thought they collaborated better. Our finding is different from the results reported in [22], which explored collaboration with mobile tablets. One reason for this difference is because the displays are relatively large, participants may have felt that the workspace was too exposed to their partner and they could do more private exploration of their own ideas. Being in B-B, users could have more privacy for exploring their ideas and confirming their hypotheses. In addition, they were still close enough to each other in this position, which allowed them to easily talk with each other or turn their face around to watch the operations performed by their partner if necessary. That is, they could have their own private workspace and still be able to discuss with the partner easily. One participant said that “I can complete the task at a private space and at the same time, we can also discuss the task just by turning the body or head”. Another participant mentioned that “My partner, if he wanted, he could watch what I am doing at any time. This feeling of being monitored was not very good”. In general, participants said that they felt safer and had more flexibility and freedom in B-B. Besides, they also said that “Being in B-B, we can pay more attention to own process of exploration”. Particularly, we also found that users in B-B gave a significantly higher score on the Satisfactory and Exploration subscales. These results also supported by participants’ comments. Based on this, it seems that by having higher feelings of satisfaction and exploration in B-B, participants were more engaged in the exploration process and were quicker at finding possible answers to the tasks. Therefore, the B-B position led to shorter exploration times.

In terms of participants’ preference on the position arrangement, the B-B position was also favored. In general, participants said that this position had two important features: (1) allowing them to have more privacy to test the ideas first without being seen by others and (2) letting them be very close to their partner. One study mentioned that the side-by-side position required less directed visual contact than other arrangements, which can improve efficiency and may be beneficial to less-talkative users [76]. However, they only explored the side-by-side and face-to-face position arrangements, and did not involve the back-to-back position. Our findings suggest that B-B was the most favorite position overall, while S-S was the second-most preferred position.

It is worth mentioning that participants’ ratings on engagement level for each position is the same as their preferred choice of position (i.e., B-B, S-S, F-F and C-C). This order is broadly aligned with the performance results. In order words, both subjective rankings on and preferences of the positions maps with performance. These observations show a strong effect of position arrangement on task performance and user experience when multiple displays and workspaces are involved.

### 6.4. Collaborative Modes and Behaviors

As stated earlier, we gave participants free choice to switch between the two interactive modes when completing the tasks. Based on our results, nine pairs chose the shared mode at all times, and five pairs chose the non-shared mode throughout the whole experiment period. The rest (16 pairs) chose to switch between the two modes (nine pairs using shared more often and seven pairs using the non-shared mode more often). From this result, it seems that the ability to switch back-and-forth was useful and preferred. It allowed participants to have a shared control and view to share ideas among multiple applications, and also have a personal space to do their own exploration independently. This way led to fewer interruptions and less distraction from their partner. Overall, more pairs (18 pairs) were in the Shared mode longer than in the non-shared mode, which aligns with **H4**. Most of users thought shared control and view would be convenient for sharing their ideas and actions with the visual elements. In addition, we also observed that the shared type of collaborative mode in large displays could enhance collaboration and communication than the non-shared mode in learning settings.

For the S-S position, more users chose the NS and NSN modes. Because of the near distance between them in S-S, these participants did not find the shared mode necessary. Instead, they thought that the non-share mode gave them better control. If they wanted to, they could turn their faces just a little and be able to see their partner’s workspace. On the other hand, more participants chose the S and SNS modes for the other positions instead of NS and NSN modes. For participants in the B-B position, none choose the NS and NSN modes. Because they usually cannot see each other without turning their heads and bodies, they preferred to keep in the S and SNS modes when completing collaborative tasks. These results also align with and support **H4** and, therefore, it can be accepted.

While in shared mode, we observed that one reason for the paired participants to speak more was that they tended to explain their actions and intentions to their partners. Although they actively shared ideas with each other, they also interacted with their own devices most of the time. In non-shared mode, we found that their communication was not as fluid as the pairs who were in shared mode. It was common for them to see the manipulations performed by the partner on the partner’s display when they communicated with each other. Sometimes they directly manipulated the visual objects on their partners’ display to demonstrate their thinking and reasoning process. For pairs choosing to switch modes, when they wanted to discuss about their ideas, they would choose shared mode. After sharing, they would explore with the tool in non-shared mode again. This suggested that these participants wanted to have some control of their interaction. The following two comments lend support to this observation: “Collaborating is fun, but sometimes I have to wait for my partner to finish,” and “It could be useful if I can choose to share my interactions or not”. Therefore, providing the function to enable switching between interactive modes would be helpful to improve users’ experience during collaboration.

### 6.5. Recommendations

The above results allow us to distill the following four recommendations for co-located learning settings with large displays when completing visual exploratory tasks.

- When considering the number of workspaces to provide to users, if the goal is to improve learning efficiency and engagement level in collaborative tasks, sharing one workspace could be chosen to support such learning tasks. This could be beneficial for improving task efficiency in collaborative learning settings. However, if the goal is to support knowledge acquisition, using separate workspaces might be a better choice.
- When considering the number of displays to provide to users, allowing multiple users (e.g., as the pairs in our study) to have a shared display would lead to better learning outcomes and engagement level rather than providing multiple separate displays during collaboration.
- When considering the position arrangements to give to users, the Back-to-Back position would provide them with higher performance and engagement levels on collaborative learning tasks. Instead, the corner-to-corner position might be not beneficial in this type of scenario.
- When considering the collaborative modes (shared and non-shared interactions), it is useful to provide a function for allowing users to freely choose whether to share or not to share the view/control of their workspace during the collaboration process. Regardless of their position arrangements, it helps to enhance users’ engagement level and the exploration process considering visual information.

## 7. Limitations and Future Work

There are several limitations in this research which can also serve to frame future work. First, while our sample size is relatively large and led to us find some significant results, a larger number of participants could have allowed us to find further insights and may be needed if we are to explore other factors and conditions that were not investigated in our study. It is also possible that with more participants we may be able to detect further significant differences in certain aspects. Despite this limitation, our results still led us to several useful findings and observations that have some practical implications. Further studies are still needed to understand the effect of more factors on task performance and user experience. In the future, we plan to use a larger sample population when the situation for large-scale studies is practical and feasible. We also plan to extend our experiment with pre-university students because it may be helpful to see if we observe similar results with younger populations like primary or high school students who, unlike university students, may have a lower level of mathematics background [59].

Second, as with other similar studies, our experiment was conducted in a lab setting. Given the potential use of the tool in educational settings, it will be helpful to have the investigation done in a more natural, in situ setting (such as a classroom). This would allow us to explore other factors that may affect the use of the tool. Also, our user study has focused on a co-located learning setting. Our findings may not apply to situations where users need to perform visual analytics tasks in remote collaboration. Given the impact of COVID-19 on how learning occurs at all levels of education, it will be relevant and valuable to see if having learners working together but remotely at separate locations would lead to the same or to different findings.

Finally, groupware technology is developing rapidly and now includes devices like virtual reality [3,7,77] and augmented reality [10,78] that have shown positive collaborative benefits. On the other hand, there is limited research on the integration of the various groupware devices into one single platform for collaborative exploration and knowledge acquisition. In the future, we plan to explore how we can integrate extended or mixed reality devices (including augmented and virtual reality) and tablets (or similar mobile technologies) with large displays to see how their coupling could benefit collaborative interaction with visual representations. Such explorations could help frame cross-device integrated environments to support collaborative learning scenarios. To develop such environments, designers and researchers need to consider the inherent limitations and affordances of these extended reality devices, including object pointing and selection issues [79], tracking and boundary of interaction [80], manipulating 3D virtual objects [81], and other interaction and feedback modalities [82,83], and tablets (e.g., [84,85]) when they are integrated together in one collaborative learning ecosystem.

## 8. Conclusions

In this research, we have investigated the use of large interactive displays to support visual collaborative learning tasks. In particular, we have explored the effect of four factors (number of virtual workspaces, number of physical displays, position arrangements of collaborators, and collaborative modes) on users task performance, engagement level, user behaviors and preferences. In this paper, we have reported the results of a user study of an interactive tool that supports the visual exploration of the properties of 3D shapes. Our results show that (1) paired users sharing one shared workspace in a large display would benefit their task efficiency and engagement level. (2) One single display would have better learning outcomes than multiple displays when completing a set of visual analytical tasks collaboratively. (3) Additionally, users placed in a back-to-back position had better task performance and greater perceived engagement levels during collaboration. (4) Finally, results show that the shared view and control or allowing users to switch between sharing and non-sharing modes provides good support for collaborative explorations that emphasize visual content using large displays. Overall, from these results, we can conclude that sharing one workspace, sharing one display, assigning users to a back-to-back position, and providing shared collaborative modes could be suitable choices for applications supporting visual exploration in collaborative settings.

## Figures and Tables

**Table 1:** Overall descriptive data for all 72 participants.

Display	Workspace	Position	Participants
1	1	S-S	12 (6 pairs)
	2	S-S	12 (6 pairs)
2	2	S-S	12 (6 pairs)
	2	F-F	12 (6 pairs)
	2	C-C	12 (6 pairs)
	2	B-B	12 (6 pairs)

**Note**: S-S: Side-by-Side; F-F: Face-to-Face; C-C: Corner-to-Corner; B-B: Back-to-Back.

**Table 2:** Summary of main statistical results.

Measurement	Variable	Key Results	*p*-Value (ANOVA)		
			Workspace	Display	Position
Performance metrics	Test improvement	–	insig.	insig.	insig.
	Time on collaborative tasks	* One single display might be useful than multiple displays for improving learning outcomes during collaboration (**P = 0.047**). * B-B got higher scores than C-C (**P = 0.045**).	insig.	**0.047**	**0.032**
	Score on collaborative tasks	–	insig.	insig.	insig.
Subjective surveys	Engagement level	* B-B is better than C-C (**P = 0.041**).	insig.	insig.	**0.044**
	*- Collaboration*	* B-B is better than C-C (P = 0.012).	insig.	insig.	**0.009**
	*- Satisfaction*	* One shared workspace might be more satisfactory than separate workspaces (**P = 0.066**). * B-B is better than C-C (**P = 0.042**).	**0.066**	insig.	**0.037**
	*- Exploration*	* B-B is better than C-C (**P = 0.025**). * B-B is better than F-F (**P = 0.038**).	insig.	insig.	**0.016**
	*- Communication*	–	insig.	insig.	insig.
	*- Comfort*	–	insig.	insig.	insig.
	*- Attention*	–	insig.	insig.	**0.054**
	*- Contribution*	–	insig.	insig.	insig.
User preference	Position arrangement	* B-B (38.33%) was the most popular choice.	–	–	–
	Collaborative mode	* More participants preferred switching between the two modes (53%). * Shared mode was preferred choice (60%).	–	–	–

**Note**: F-F: Face-to-Face; C-C: Corner-to-Corner; B-B: Back-to-Back. Bold fonts represent statistically significant results.

**Table 3:** Main mean results of three variables.

Variables		Time/mins	Score/Points
**Workspace**	One	M = 21.833, SD = 4.997, SE = 2.040	M = 9.00, SD = 2.098, SE = 0.856
	Two	M = 24.500, SD = 7.176, SE = 2.930	M = 9.67, SD = 1.033, SE = 0.422
**Display**	One	M = 24.833, SD = 7.731, SE = 3.156	M = 9.667, SD = 1.033, SE = 0.422
	Two	M = 27.500, SD = 8.093, SE = 3.304	M = 8.500, SD = 0.837, SE = 0.342
**Position**	S-S	M = 24.333, SD = 3.033, SE = 1.647	M = 8.500, SD = 0.837, SE = 0.342
	F-F	M = 25.500, SD = 3.891, SE = 2.405	M = 7.500, SD = 1.225, SE = 0.500
	C-C	M = 28.500, SD = 4.432, SE = 2.442	M = 6.833, SD = 1.602, SE = 0.654
	B-B	M = 23.167, SD = 2.834, SE = 1.977	M = 9.000, SD = 1.265, SE = 0.298

**Note**: S-S: Side-by-Side; F-F: Face-to-Face; C-C: Corner-to-Corner; B-B: Back-to-Back.

**Table 4:** Participants’ preference on collaboration modes (based on pairs of participants).

Condition	S Mode	NS Mode	NSS Mode	SNS Mode
One display with one workspace (S-S)	-	-	-	-
One display with two workspaces (S-S)	0	4	2	0
Two displays with separate workspace (S-S)	0	1	3	2
Two displays with separate workspace (F-F)	2	0	1	3
Two displays with separate workspace (C-C)	3	0	1	2
Two displays with separate workspace (B-B)	4	0	0	2
Overall	9	5	7	9
	14		16	

**Note**: S: Shared mode at all times; NS: Non-shared mode at all times; NSS: more Non-shared time when switching between modes; SNS: more Shared time when switching between modes. S-S: Side-by-Side; F-F: Face-to-Face; C-C: Corner-to-Corner; B-B: Back-to-Back.

## Data Availability

The datasets used and/or analyzed during the current study are available from the corresponding author on reasonable request.

## Abbreviations

The following abbreviations are used in this manuscript:

CSCL	Computer-supported collaborative learning
VR	Virtual Reality
AR	Augmented Reality
STM	Solid transition maps
S-S	Side-by-Side
F-F	Face-to-Face
C-C	Corner-to-Corner
B-B	Back-to-Back
M	mean
SD	standard deviation
SE	standard error
ANOVA	analysis of variance

## References

[1] Bruffee K.A. (1984). Collaborative learning and the “conversation of mankind”. Coll. Engl..

[2] Dillenbourg P. (1999). What do You Mean by Collaborative Learning?. Collaborative Learning: Cognitive and Computational Approaches.

[3] Chen L., Liang H.N., Lu F., Wang J., Chen W., Yue Y. (2021). Effect of Collaboration Mode and Position Arrangement on Immersive Analytics Tasks in Virtual Reality: A Pilot Study. Appl. Sci..

[4] Laal M., Ghodsi S.M. (2012). Benefits of collaborative learning. Procedia-Soc. Behav. Sci..

[5] Cen L., Ruta D., Powell L., Hirsch B., Ng J. (2016). Quantitative approach to collaborative learning: Performance prediction, individual assessment, and group composition. Int. J. Comput.-Support. Collab. Learn..

[6] Al-Rahmi W.M., Zeki A.M. (2017). A model of using social media for collaborative learning to enhance learners’ performance on learning. J. King Saud Univ.-Comput. Inf. Sci..

[7] Liang H.N., Lu F., Shi Y., Nanjappan V., Papangelis K. (2019). Evaluating the effects of collaboration and competition in navigation tasks and spatial knowledge acquisition within virtual reality environments. Future Gener. Comput. Syst..

[8] Zagermann J., Pfeil U., Rädle R., Jetter H.C., Klokmose C., Reiterer H. When tablets meet tabletops: The effect of tabletop size on around-the-table collaboration with personal tablets. Proceedings of the 2016 CHI Conference on Human Factors in Computing Systems.

[9] Jakobsen M.R., Hornbæk K. (2014). Up close and personal: Collaborative work on a high-resolution multitouch wall display. ACM Trans. Comput.-Hum. Interact. (TOCHI).

[10] Chen L., Liu Y., Li Y., Yu L., Gao B., Caon M., Yue Y., Liang H.N. Effect of visual cues on pointing tasks in co-located augmented reality collaboration. Proceedings of the ACM Symposium on Spatial User Interaction (SUI).

[11] Scott S.D., Grant K.D., Mandryk R.L. (2003). System Guidelines for Co-Located, Collaborative Work on a Tabletop Display.

[12] Vogt K., Bradel L., Andrews C., North C., Endert A., Hutchings D. (2011). Co-located collaborative sensemaking on a large high-resolution display with multiple input devices. IFIP Conference on Human-Computer Interaction.

[13] Paul C.L., Bradel L. Size matters: The effects of interactive display size on interaction zone expectations. Proceedings of the 2018 International Conference on Advanced Visual Interfaces—AVI’18, Castiglione della Pescaia.

[14] Kruger R., Carpendale S., Scott S.D., Greenberg S. (2004). Roles of orientation in tabletop collaboration: Comprehension, coordination and communication. Comput. Support. Coop. Work (CSCW).

[15] Alallah F., Jin D., Irani P. OA-graphs: Orientation agnostic graphs for improving the legibility of charts on horizontal displays. Proceedings of the ACM International Conference on Interactive Tabletops and Surfaces—ITS’10.

[16] Isenberg P., Fisher D., Paul S.A., Morris M.R., Inkpen K., Czerwinski M. (2011). Co-located collaborative visual analytics around a tabletop display. IEEE Trans. Vis. Comput. Graph..

[17] Liu C.C., Kao L.C. (2007). Do handheld devices facilitate face-to-face collaboration? Handheld devices with large shared display groupware to facilitate group interactions. J. Comput. Assist. Learn..

[18] Sinha S., Rogat T.K., Adams-Wiggins K.R., Hmelo-Silver C.E. (2015). Collaborative group engagement in a computer-supported inquiry learning environment. Int. J. Comput.-Support. Collab. Learn..

[19] Guillomía M.A., Artigas J.I., Falcó J.L. (2021). Cognitive Accessibility and Support in Special Education. Sensors.

[20] Praharaj S., Scheffel M., Schmitz M., Specht M., Drachsler H. (2021). Towards automatic collaboration analytics for group speech data using learning analytics. Sensors.

[21] Garcia-Sanjuan F., Jurdi S., Jaen J., Nacher V. (2018). Evaluating a tactile and a tangible multi-tablet gamified quiz system for collaborative learning in primary education. Comput. Educ..

[22] Chen L., Liang H.N., Lu F., Papangelis K., Man K.L., Yue Y. (2020). Collaborative behavior, performance and engagement with visual analytics tasks using mobile devices. Hum.-Cent. Comput. Inf. Sci..

[23] Tissenbaum M., Berland M., Lyons L. (2017). DCLM framework: Understanding collaboration in open-ended tabletop learning environments. Int. J. Comput.-Support. Collab. Learn..

[24] Cardoso J., Ribeiro J.M. (2021). Tangible VR Book: Exploring the Design Space of Marker-Based Tangible Interfaces for Virtual Reality. Appl. Sci..

[25] Yotam H., Twersky D. (2020). Distributed spatial Sensemaking on the augmented reality sandbox. Int. J. Comput.-Support. Collab. Learn..

[26] Czerwinski M., Robertson G., Meyers B., Smith G., Robbins D., Tan D. Large display research overview. Proceedings of the 2006 CHI Conference Extended Abstracts on Human Factors in Computing Systems—CHI EA.

[27] Butscher S., Hubenschmid S., Müller J., Fuchs J., Reiterer H. Clusters, trends, and outliers: How immersive technologies can facilitate the collaborative analysis of multidimensional data. Proceedings of the 2018 CHI Conference on Human Factors in Computing Systems—CHI’16.

[28] Sharma K., Leftheriotis I., Giannakos M. (2020). Utilizing Interactive Surfaces to Enhance Learning, Collaboration and Engagement: Insights from Learners’ Gaze and Speech. Sensors.

[29] Bradel L., Endert A., Koch K., Andrews C., North C. (2013). Large high resolution displays for co-located collaborative sensemaking: Display usage and territoriality. Int. J. Hum.-Comput. Stud..

[30] Wallace J.R., Scott S.D., MacGregor C.G. Collaborative sensemaking on a digital tabletop and personal tablets: Prioritization, comparisons, and tableaux. Proceedings of the SIGCHI Conference on Human Factors in Computing Systems—CHI’13.

[31] Bause I.M., Brich I.R., Wesslein A.K., Hesse F.W. (2018). Using technological functions on a multi-touch table and their affordances to counteract biases and foster collaborative problem solving. Int. J. Comput.-Support. Collab. Learn..

[32] Kharrufa A., Leat D., Olivier P. Digital mysteries: Designing for learning at the tabletop. Proceedings of the ACM International Conference on Interactive Tabletops and Surfaces—ITS’10.

[33] Antle A.N., Bevans A., Tanenbaum T.J., Seaborn K., Wang S. Futura: Design for collaborative learning and game play on a multi-touch digital tabletop. Proceedings of the Fifth International Conference on Tangible, Embedded, and Embodied Interaction—TEI’10.

[34] Schnaubert L., Bodemer D. (2019). Providing different types of group awareness information to guide collaborative learning. Int. J. Comput.-Support. Collab. Learn..

[35] Martinez-Maldonado R. (2019). A handheld classroom dashboard: Teachers’ perspectives on the use of real-time collaborative learning analytics. Int. J. Comput.-Support. Collab. Learn..

[36] Dillon A., Richardson J., McKnight C. (1990). The effects of display size and text splitting on reading lengthy text from screen. Behav. Inf. Technol..

[37] Bruijn D.D., Mul S.D., Oostendorp H.V. (1992). The influence of screen size and text layout on the study of text. Behav. Inf. Technol..

[38] Czerwinski M., Smith G., Regan T., Meyers B., Robertson G.G., Starkweather G.K. (2003). Toward characterizing the productivity benefits of very large displays. Interact.

[39] Mandryk R.L., Scott S.D., Inkpen K.M. (2002). Display factors influencing co-located collaboration. Comference Suppl. ACM CSCW.

[40] Strijbos J.W. (2010). Assessment of (computer-supported) collaborative learning. IEEE Trans. Learn. Technol..

[41] Bly S.A. A use of drawing surfaces in different collaborative settings. Proceedings of the 1988 ACM Conference on Computer-Supported Cooperative Work—CSCW’88.

[42] Inkpen K., Hawkey K., Kellar M., Mandryk R., Parker K., Reilly D., Scott S., Whalen T. (2005). Exploring display factors that influence co-located collaboration: Angle, size, number, and user arrangement. Proc. HCI Int..

[43] Hawkey K., Kellar M., Reilly D., Whalen T., Inkpen K.M. The proximity factor: Impact of distance on co-located collaboration. Proceedings of the 2005 International ACM SIGGROUP Conference on Supporting Group Work—CSCW’05.

[44] Tang A., Tory M., Po B., Neumann P., Carpendale S. Collaborative coupling over tabletop displays. Proceedings of the SIGCHI Conference on Human Factors in Computing Systems—CHI’06.

[45] Liu C., Chapuis O., Beaudouin-Lafon M., Lecolinet E. Shared interaction on a wall-sized display in a data manipulation task. Proceedings of the 2016 CHI Conference on Human Factors in Computing Systems—CHI’16.

[46] Ha V., Inkpen K.M., Mandryk R.L., Whalen T. Direct intentions: The effects of input devices on collaboration around a tabletop display. Proceedings of the First IEEE International Workshop on Horizontal Interactive Human-Computer Systems— ITS’06.

[47] Balakrishnan A.D., Fussell S.R., Kiesler S. Do visualizations improve synchronous remote collaboration?. Proceedings of the SIGCHI Conference on Human Factors in Computing Systems—CHI’08.

[48] Greenberg S. (1990). Sharing views and interactions with single-user applications. ACM SIGOIS Bull..

[49] Isenberg P., Elmqvist N., Scholtz J., Cernea D., Ma K.L., Hagen H. (2011). Collaborative visualization: Definition, challenges, and research agenda. Inf. Vis..

[50] Huang Z., Zhu Y., Mao X., Su T., Fu X., Fei G. coisTable: An Individual-and-Spatial-Aware Tabletop System for Co-located Collaboration. Proceedings of the 2020 6th International Conference on Dependable Systems and Their Applications—DSA’20.

[51] Chung H., North C. (2018). SAViL: Cross-display visual links for sensemaking in display ecologies. Pers. Ubiquitous Comput..

[52] Kraut R.E., Gergle D., Fussell S.R. The use of visual information in shared visual spaces: Informing the development of virtual co-presence. Proceedings of the 2002 ACM Conference on Computer Supported Cooperative Work—CSCW’02.

[53] Larsen-Ledet I., Korsgaard H. (2019). Territorial functioning in collaborative writing. Comput. Support. Coop. Work (CSCW).

[54] Scott S.D., Carpendale M.S.T., Inkpen K. Territoriality in collaborative tabletop workspaces. Proceedings of the 2004 ACM Conference on Computer Supported Cooperative Work—CSCW’04.

[55] Xu Y., Wang L., Xu Y., Qiu S., Xu M., Meng X. (2019). Cross-device task interaction framework between the smart watch and the smart phone. Pers. Ubiquitous Comput..

[56] Russell D.M., Drews C., Sue A. (2002). Social aspects of using large public interactive displays for collaboration. International Conference on Ubiquitous Computing.

[57] Russell D.M. (2003). Large interactive public displays: Use patterns, support patterns, community patterns. Public, Community and Situated Displays.

[58] Phelps E., Damon W. (1989). Problem solving with equals: Peer collaboration as a context for learning mathematics and spatial concepts. J. Educ. Psychol..

[59] Liang H.N., Sedig K. (2010). Can interactive visualization tools engage and support pre-university students in exploring non-trivial mathematical concepts?. Comput. Educ..

[60] Sedig K., Liang H.N. (2008). Learner-information interaction: A macro-level framework characterizing visual cognitive tools. J. Interact. Learn. Res..

[61] Liang H.N., Sedig K. (2010). Role of interaction in enhancing the epistemic utility of 3D mathematical visualizations. Int. J. Comput. Math. Learn..

[62] Lu F., Yu D., Liang H.N., Chen W., Papangelis K., Ali N.M. Evaluating Engagement Level and Analytical Support of Interactive Visualizations in Virtual Reality Environments. Proceedings of the 2018 IEEE International Symposium on Mixed and Augmented Reality—ISMAR’18.

[63] Unity Unity Real-Time Development Platform|3D, 2D VR & AR Engine. https://unity.com/.

[64] Sedig K., Rowhani S., Liang H.N. (2005). Designing interfaces that support formation of cognitive maps of transitional processes: An empirical study. Interact. Comput..

[65] Ballas J.A., Heitmeyer C.L., Pérez-Quiñones M.A. Evaluating two aspects of direct manipulation in advanced cockpits. Proceedings of the SIGCHI Conference on Human Factors in Computing Systems—CHI’92.

[66] Sedig K., Parsons P., Liang H.N., Morey J. (2016). Supporting sensemaking of complex objects with visualizations: Visibility and complementarity of interactions. Informatics.

[67] Sedig K., Liang H.N. (2006). Interactivity of Visual Mathematical Representations: Factors Affecting Learning and Cognitive Processes. J. Interact. Learn. Res..

[68] Roberts J.C. State of the art: Coordinated & multiple views in exploratory visualization. Proceedings of the Fifth International Conference on Coordinated and Multiple Views in Exploratory Visualization—CMV’07.

[69] Carini R.M., Kuh G.D., Klein S.P. (2006). Student engagement and student learning: Testing the linkages. Res. High. Educ..

[70] Christenson S.L., Reschly A.L., Wylie C. (2012). Handbook of Research on Student Engagement.

[71] Hung Y.H., Parsons P. Assessing user engagement in information visualization. Proceedings of the 2017 CHI Conference Extended Abstracts on Human Factors in Computing Systems—CHI’17.

[72] Wallace J.R., Iskander N., Lank E. Creating your bubble: Personal space on and around large public displays. Proceedings of the 2016 CHI Conference on Human Factors in Computing Systems—CHI’16.

[73] Buisine S., Besacier G., Aoussat A., Vernier F. (2012). How do interactive tabletop systems influence collaboration?. Comput. Hum. Behav..

[74] Chan C.K., Chan Y.Y. (2011). Students’ views of collaboration and online participation in Knowledge Forum. Comput. Educ..

[75] O’hara K., Sellen A. A comparison of reading paper and on-line documents. Proceedings of the ACM SIGCHI Conference on Human Factors in Computing Systems—CHI’97.

[76] Rodden T., Rogers Y., Halloran J., Taylor I. Designing novel interactional workspaces to support face to face consultations. Proceedings of the SIGCHI Conference on Human Factors in Computing Systems—CHI’03.

[77] Martikainen S., Wikström V., Falcon M., Saarikivi K. Collaboration face-to-face and in virtual reality-Empathy, social closeness, and task load. Proceedings of the Conference Companion Publication of the 2019 on Computer Supported Cooperative Work and Social Computing.

[78] Ens B., Lanir J., Tang A., Bateman S., Lee G., Piumsomboon T., Billinghurst M. (2019). Revisiting collaboration through mixed reality: The evolution of groupware. Int. J. Hum.-Comput. Stud..

[79] Yu D., Liang H.N., Lu X., Fan K., Ens B. (2019). Modeling Endpoint Distribution of Pointing Selection Tasks in Virtual Reality Environments. ACM Trans. Graph. (TOG).

[80] Xu W., Liang H.N., Chen Y., Li X., Yu K. Exploring Visual Techniques for Boundary Awareness During Interaction in Augmented Reality Head-Mounted Displays. Proceedings of the 2020 IEEE Conference on Virtual Reality and 3D User Interfaces—VR’20.

[81] Nanjappan V., Liang H.N., Lu F., Papangelis K., Yue Y., Man K. (2018). User-elicited dual-hand interactions for manipulating 3D objects in virtual reality environments. Hum. Cent. Comput. Inf. Sci..

[82] Yu D., Lu X., Shi R., Liang H.N., Dingler T., Velloso E., Goncalves J. Gaze-Supported 3D Object Manipulation in Virtual Reality. Proceedings of the 2021 CHI Conference on Human Factors in Computing Systems—CHI’21.

[83] Monteiro D., Liang H.N., Wang X., Xu W., Tu H. Design and Development of a Low-Cost Device for Weight and Center of Gravity Simulation in Virtual Reality. Proceedings of the 2021 International Conference on Multimodal Interaction—ICMI’21.

[84] Liang H.N., Williams C., Semegen M., Stuerzlinger W., Irani P. User-Defined Surface+motion Gestures for 3d Manipulation of Objects at a Distance through a Mobile Device. Proceedings of the 10th Asia Pacific Conference on Computer Human Interaction—APCHI’12.

[85] Liang H.N., Williams C., Semegen M., Stuerzlinger W., Irani P. (2013). An investigation of suitable interactions for 3D manipulation of distant objects through a mobile device. Int. J. Innov. Comput. Inf. Control.

